# Using microbial metalo-aminopeptidases as targets in human infectious diseases

**DOI:** 10.15698/mic2021.10.761

**Published:** 2021-08-09

**Authors:** Jorge González-Bacerio, Maikel Izquierdo, Mirtha Elisa Aguado, Ana C. Varela, Maikel González-Matos, Maday Alonso del Rivero

**Affiliations:** 1 Center for Protein Studies, Faculty of Biology, University of Havana, calle 25 #455 entre I y J, 10400, Vedado, La Habana, Cuba.; 2 Department of Biochemistry, Faculty of Biology, University of Havana, calle 25 #455 entre I y J, 10400, Vedado, La Habana, Cuba.

**Keywords:** microbial metalo-aminopeptidases, molecular targets, human infectious diseases

## Abstract

Several microbial metalo-aminopeptidases are emerging as novel targets for the treatment of human infectious diseases. Some of them are well validated as targets and some are not; some are essential enzymes and others are important for virulence and pathogenesis. For another group, it is not clear if their enzymatic activity is involved in the critical functions that they mediate. But one aspect has been established: they display relevant roles in bacteria and protozoa that could be targeted for therapeutic purposes. This work aims to describe these biological functions for several microbial metalo-aminopeptidases.

## INTRODUCTION

Several microbial metalo-aminopeptidases are emerging as novel targets for the treatment of human infectious diseases. Some of them are well validated as targets and some are not; some are essential enzymes and others are important for virulence and pathogenesis. For another group, it is not clear if their enzymatic activity is involved in the critical functions that they mediate. But one aspect has been established: they display relevant roles in bacteria and protozoa that could be targeted for therapeutic purposes. This work aims to describe these biological functions for several microbial metalo-aminopeptidases. The main biological functions and molecular properties of these enzymes that support them as targets are presented in **Table 1**.

**Table 1:** Main biological functions and molecular properties of microbial metalo-aminopeptidases that support their essentiality or involvement in virulence.

**Protease family**	**Metalo-aminopeptidase (source)**	**Molecular activity or property that determines their main functions**	**Main functions**	**Essentiality or involvement in virulence**	**Experimental evidences that support their relevance**
M1	PfA-M1 (*P. falciparum* parasite)	Alanyl-aminopeptidase activity	Hemoglobin degradation	Essential	- Bestatin and specific inhibitors block parasite growth - Their toxicity is reduced in parasites overex-pressing PfA-M1 - Knockout is lethal
M17	*Mt*LAP (*M. tuberculosis* bacterium)	Leucyl-aminopeptidase activity	Unknown	Essentiality not demonstrated	Bestatin inhibits bacterial growth *in vitro* and during macrophage infection
	PfA-M17 (*P. falciparum* parasite)	Leucyl-aminopeptidase activity	- Hemoglobin degradation - Erythrocyte invasion (probably) - Other housekeeping functions	Essential	- Bestatin and a specific inhibitor block parasite growth - Knockout is lethal
	*Tb*LAP-B (*T. brucei* parasite)	Leucyl-aminopeptidase activity (probably)	Kinetoplast DNA segregation	Not essential, involved in virulence	Down-regulation induces a delay in cytokinesis
	LAP-B (*Leishmania* spp. parasite)	Leucyl-aminopeptidase activity (probably)	- Leucine supply during host infection - Intracellular protein degradation and turnover - Host cell invasion (all probably)	Essentiality not demonstrated	Selective inhibition may interfere with parasite viability
	*Ac*LAP (*A. castellanii* parasite)	Leucyl-aminopeptidase activity	Encystation	Not essential, involved in virulence	Knockdown and bestatin produce encystation inhibition
	*Tg*LAP (*T. gondii* parasite)	Leucyl-aminopeptidase activity (probably)	Hydrolysis of dipeptides produced by cathepsin Cs and pro-teasoma (probably)	Not essential, involved in virulence	Knockout inhibits the parasite ability to attach and/or invade cultured cells, attenuating virulence in a mouse model
	*Sa*M17-LAP (*S. aureus* bacterium)	Cysteinyl-glycinase activity (probably)	- Bioactivates / inactivates key cellular proteins involved in metabolism, cell wall biosynthesis or signaling - Sulfur metabolism (all probably)	Not essential, involved in virulence	- Required *in vitro* for bacterial survival inside human macrophages - Knockout attenuates virulence in *in vivo* mouse models
M17	*Td*M17-LAP (*T. denticola* bacterium)	Cysteinyl-glycinase activity	Glutathione catabolic pathway	Essentiality not demonstrated	Glutathione and Cys-Gly protect the cellular components from oxidative damage
	*Hp*M17AP (*H. pylori* bacterium)	Cysteinyl-glycinase and arginyl-aminopeptidase activities	- Defense in human macrophages - Drug resistance mechanisms - Housekeeping role - Maintains an adequate cytoplasmic pool of free arginine (all probably)	Essentiality not demonstrated	- Upregulated in response to the anti-*H. pylori agent*, NE-2001, and oxidative stress caused by nitric oxide and metronidazole - Bestatin inhibits bacterial growth
	PhpA (*P. aeruginosa* bacterium)	Quaternary structure	Regulates transcription of the virulence-associated *algD* gene	Not essential, involved in virulence	Mutation in a metal-binding residue increases transcription of *algD* gene and produces a slow growth phenotype *in vivo*
	*Vc*PepA (*V. cholera* bacterium)	Quaternary structure	Modulates transcription of the cholera toxin gene under different environmental conditions	Not essential, involved in virulence	Knockout increases levels of cholera toxin in non-inducing conditions
M18	PfA-M18 (*P. falciparum* parasite)	Aspartyl-aminopeptidase activity	- Hemoglobin degradation - Erythrocyte membrane rupture (all probably)	Essential	Knockdown inhibits parasite growth
M24	*Mt*MetAP1a (*M. tuberculosis* bacterium)	Methionyl-aminopeptidase activity	Removal of N-terminal methio-nine from newly synthesized peptides	Essential	- Knockdown inhibits bacterial growth - Overexpression confers resistance to the antibacterial effect of enzymatic inhibitors
	*Mt*MetAP1c (*M. tuberculosis* bacterium)	Methionyl-aminopeptidase activity	- Removal of N-terminal methionine from newly synthesized peptides - A major role in the host macrophage phagosome	Not essential, involved in virulence	Overexpression confers resistance to the antibacterial effect of enzymatic inhibitors
	*Ld*MetAP2 (*L. donovani* parasite)	Methionyl-aminopeptidase activity	- Apoptosis - Removal of N-terminal methionine from newly synthesized peptides	Essentiality not demonstrated	- Overexpression and apoptosis are associated - Inhibitors prevent the induction of apoptosis, but do not prevent parasite death

## AMINOPEPTIDASES BELONGING TO THE M1 FAMILY OF PROTEASES

The M1 alanyl-aminopeptidase from the parasite *Plasmodium falciparum* (PfA-M1) is involved in hemoglobin degradation (an essential process [1]) during erythrocytic stages [2–4], when the parasite catabolizes 65-75 % of host hemoglobin [5]. This event guarantees vital space for parasite growth inside the erythrocyte [6], generates free amino acids for parasite protein synthesis [7, 8], modulates osmotic pressure within infected red blood cells, prevents premature erythrocyte lysis [9] and guarantees the uptake of extracellular isoleucine (an essential amino acid absent in human hemoglobin [7, 10]) through exchange with intracellular leucine [11, 12].

It has been proposed that PfA-M1 develops crucial functions to the parasite life cycle [3], being the main evidences: (1) The *in vivo* parasite growth is inhibited by bestatin, a classical inhibitor of many metaloaminopepti-dases, and compound 4, a synthetic PfA-M1 inhibitor [13], in the murine malaria model *Plasmodium chabaudi* [14]. (2) The toxicity of these compounds is reduced in transgenic parasites overexpressing PfA-M1 [13]. (3) The PfA-M1 specific inhibitors inhibit the *in vitro* parasite growth [4]. (4) The absence of this enzyme in knockout parasites is lethal [2]. All of these results are indicative of the target character of PfA-M1 for the search of a new class of antimalarials [3, 4].

## AMINOPEPTIDASES BELONGING TO THE M17 FAMILY OF PROTEASES

M17 aminopeptidases have leucyl-aminopeptidase (LAP) activity, responsible in most cases for the biological functions related with their target character. But other M17 enzymes, mainly from bacteria, exhibit also cysteinyl-glycinase activity, which is involved in their critical cellular functions. Another group of M17 LAPs have roles that do not depend on their enzymatic activity, but on their quaternary structure (transcriptional regulation, for example).

### M17 aminopeptidases whose main function depends on LAP activity

#### M17 LAP from the bacterium Mycobacterium tuberculosis

The growth inhibition of the bacterium *M. tuberculosis* by bestatin, *in vitro* and during macrophage infection, supports the involvement of the M17 leucyl-aminopeptidase (*Mt*LAP) in physiological and pathogenic processes in tuberculosis. This enzyme is probably essential for *in vivo* bacterial survival and pathogenesis [15].

#### M17 LAP from P. falciparum

The M17 LAP from *P. falciparum* (PfA-M17) is involved in the hemoglobin digestion, with the functions described above. The blockade of this LAP activity is toxic *in vitro* for *P. falciparum* and *P. chabaudi chabaudi* [16, 17]. In contrast to PfA-M1, PfA-M17 may have additional functions, since its specific inhibition in parasite cultures causes growth retardation early in the erythrocytic stages, before hemoglobin digestion begins [4]. PfA-M17 could participate in red cell invasion process, since bestatin diminishes the rings number 24 h after addition of schizont-infected erythrocytes to uninfected cells [17]. This enzyme is essential for parasite viability, since PfA-M17 gene knockout has been unsuccessful [2].

#### Basic M17 LAP from the parasite Trypanosoma brucei

In host infection processes, the basic M17 LAP from the parasite *T. brucei* (*Tb*LAP-B) could have some of the following functions: provide an essential amino acid (leucine is a precursor for sterol biosynthesis [18, 19]), being involved in infectivity [20], regulate stress responses and signal transduction [21], act as protein chaperones [22], be required for glutathione metabolism [23], and participate in host cell invasion [24, 25].

Interference RNA-mediated down-regulation of *Tb*LAP-B induces a nonlethal growth defect, causing a delay in cytokinesis. Ectopic expression of the *Tb*LAP-B-hemaglutinin fusion in procyclic *T*. *brucei* causes the loss of kinetoplast DNA, failure of the mitochondrial membrane potential and related growth defects. Parasites expressing *Tb*LAP-B-hemaglutinin can duplicate their kinetoplast DNA, but correct separation fails. The enzyme down-regulation and ectopic expression indicate its clear involvement in kinetoplast DNA segregation [26].

#### Basic M17 LAP from the parasite Leishmania spp.

The LAP activity of the soluble extracts of the parasite *Leishmania* spp. was almost completely (90-95 %) inhibited by anti-porcine LAP IgG (this antibody inhibits the basic M17 LAPs from *Leishmania* spp. -LAP-Bs-), indicating that LAP-Bs are responsible for the bulk of this activity in parasite extracts. The selective inhibition of LAP-B may interfere with parasite viability [27], because *Leishmania* spp. are auxotrophic for branched-chain amino acids [28, 29]. Therefore, LAP-B could provide an essential amino acid in host infection processes (leucine is a precursor for fatty acids and sterol biosynthesis [18]). Furthermore, LAP-B could participate in intracellular protein degradation and turnover [30], and host cell invasion [24, 25].

#### M17 LAP from the parasite Acanthamoeba castellanii

The cysts of the parasite *A. castellanii*, knocked-down for M17 LAP (*Ac*LAP), do not show separated ectocyst and endocyst, discernible by transmission electronic microscopy, indicating cell wall rupture. A similar morphology exhibit cells treated with bestatin, suggesting that decreased *Ac*LAP activity causes parasite cell wall ultrastructural changes, closely related with encystation inhibition. It is possible that the affectation in protein turnover blocks the cyst wall synthesis or produces the cell breakdown by oligopeptide accumulation [20]. However, a selective M17 LAP inhibitor is required to confirm that this phenotype is only the result of the *Ac*LAP inhibition [31].

#### M17 LAP from the parasite Toxoplasma gondii

The M17 LAP from the parasite *T. gondii* (*Tg*LAP) could be involved in the hydrolysis of dipeptides produced by cathepsin Cs in parasitophorous vacuole [32]. Alternatively, the *Tg*LAP substrates could be peptides generated in the proteasomal protein degradation pathway [33]. Knockout of *Tg*LAP inhibits the parasite's ability to attach and/or invade cultured cells, and this reduces replication and attenuates virulence in a mouse model [34]. However, this phenotype has not been directly associated with the enzyme LAP activity, and could be related to other unknown protein functions [31].

### M17 aminopeptidases whose main function depends on cysteinyl-glycinase activity

#### M17 LAP from the bacterium Staphylococcus aureus

Despite not being essential for the bacterium *S. aureus*, its M17 LAP (*Sa*M17-LAP) plays an important role in virulence. This enzyme is required *in vitro* for bacterial survival inside human macrophages. Further, *S. aureus* with a disrupted *Sa*M17-LAP gene had severely attenuated virulence in both localized and systemic infections in *in vivo* mouse models. It has been proposed that *Sa*M17-LAP bioactivates/inactivates key cellular proteins involved in crucial functions, such as metabolism, cell wall biosynthesis or signaling. This proteolysis would confer any advantage for the bacterium in the harsh host environment [35].

*S. aureus* produces the low-molecular-weight thiol bacillithiol (Cys-GlcN-mal) instead? of glutathione [36]. Cysteine-containing molecules are cysteine sources during nutrient restriction [37], and are important in cellular defense against low pH, oxidative and osmotic stress. In addition, sulfur metabolism has been linked to virulence [38, 39]. For this reason, the cysteinyl-glycinase activity of *Sa*M17-LAP suggests its importance for *S. aureus* virulence [40].

#### M17 LAP from the bacterium Treponema denticola

The M17 LAP from the bacterium *T. denticola* (*Td*M17-LAP) was identified as the probably only cysteinyl-glycinase involved in the glutathione catabolic pathway, by immunodepletion of the most cysteinyl-glycinase activity in the soluble fraction of sonicated *T. denticola* cells, when the bacterium was grown under standard conditions. Hydrogen sulfide, ammonium, pyruvate, glutamate and glycine are produced in equimolar amounts by this pathway [23, 41]. Both glutathione and Cys-Gly can play critical roles in maintaining cellular redox status, protecting the cellular components from oxidative damage. These two thiol-containing molecules can also modify the cysteine residues of some proteins, regulating their activities [42].

#### M17 LAP from the bacterium Helicobacter pylori

The M17 LAP from the bacterium *H. pylori* (*Hp*M17AP) is upregulated in response to the anti-*H. pylori* agent, NE-2001 [43], and oxidative stress caused by nitric oxide [44] and metronidazole [45]. These evidences, together with the enzyme allosteric nature and high efficiency, suggest that *Hp*M17AP may play a relevant role in the *H. pylori* life cycle [46]. The response against nitric oxide [44] suggests a role in defense in human macrophages [47]. In addition, the response against metronidazole suggests an involvement in drug resistance mechanisms, in addition to a relevant housekeeping role [45]. These *Hp*M17AP functionalities in response to cellular oxidative stress could potentially result from the cysteinyl-glycinase activity of the protein [45, 47].

*H. pylori* utilizes the stomach's mucosal glutathione, produced as the major defense mechanism against low pH, oxidative and osmotic stress [38], as a glutamate source [48]. The resultant Cys-Gly dipeptide produced by the glutathione catabolism is cleaved to salvage cysteine [47]. On the other hand, high activity of *Hp*M17AP on peptides with essential N-terminal arginine [49] may contribute to maintain an adequate cytoplasmic pool of free arginine, which could be used for synthesis of polyamines required for optimal *H. pylori* growth [47]. Bestatin inhibits the growth of *H. pylori* in culture [46], an effect probably caused by *Hp*M17AP inhibition [31].

### M17 aminopeptidases whose main function depends on their quaternary structure

#### M17 LAP from the bacterium Pseudomonas aeruginosa

The hexameric M17 LAP from the bacterium *P. aeruginosa* (PhpA) transcriptionally regulates the virulence-associated *algD* gene, encoding an enzyme of the alginate biosynthetic pathway [50]. Alginate is involved in biofilm formation, and its overproduction characterizes the highly-mucoid phenotype of cystic fibrosis in the lung [51]. By mutating one of the PhpA metal-binding residues, but not by bestatin inhibition, the transcription of the *algD* gene is increased and a slow growth phenotype is generated *in vivo*. This suggests that the aminopeptidase activity is not required for transcriptional regulation [50], and mutations could result in hexamer disruption [31], as observed for tomato M17 LAP [52].

#### M17 LAP from the bacterium Vibrio cholera

In the bacterium *V. cholerae* the expression of virulence factors, such as cholera toxin, are mediated by a complex regulatory circuit, highly dependent on environmental temperature and pH. Disruption of the gene encoding the M17 LAP from *V. cholerae* (*Vc*PepA) resulted in increased levels of cholera toxin under non-inducing conditions (pH 8.4 and 37°C), under which toxins would normally not be observed. In contrast, under inducing conditions (pH 6.5 and 30°C), the absence of *Vc*PepA has no effect on toxin levels [53]. Behari *et al.* [53] identified a potential target sequence in the *V. cholerae* genome to which *Vc*PepA might bind, and therefore propose that the protein modulates transcription of the toxin gene under different environmental conditions. Enzymatic activity of *Vc*PepA would not be involved in this function.

## AMINOPEPTIDASES BELONGING TO the M18 FAMILY OF PROTEASES

The M18 aspartyl-aminopeptidase from *P. falciparum* (PfA-M18) could be involved in protein catabolism, including the turnover of parasite proteins and hemoglobin degradation. The parasitophorous vacuole location (in addition to cytosolic) suggests that, like PfA-M17, PfA-M18 may have other relevant functions in addition to hemoglobin digestion [54]. For example, the enzyme could have a role in erythrocyte membrane rupture during merozoite release or reinvasion, since it binds the membrane protein spectrin [55].

PfA-M18 knockdown results in a lethal phenotype with relevant morphological alterations, as was observed by electron microscopy [54]. Other gene disruption/truncation experiments, resulting in ∼10 % aspartyl-aminopeptidase activity compared to wild-type parasites, indicate that the enzyme is dispensable for the erythrocytic cycle but this generates negative consequences for the parasite [2].

## AMINOPEPTIDASES BELONGING TO THE M24 FAMILY OF PROTEASES

### M24 methionyl-aminopeptidases (MetAP) from *M. tuberculosis*

Bacterial protein synthesis is initiated with an *N*-formylmethionine, whose *N*-formyl group is removed by peptide deformylase. Thereafter, M24 methionyl-aminopeptidases (MetAPs) remove the N-terminal methionine. Since this essential process is required for protein post-translational modifications, activity, stability, localization or degradation, the excision pathway is a potential drug target in tuberculosis [56].

*M. tuberculosis* MetAP1a (*Mt*MetAP1a) antisense-RNA-knockdown, and not *Mt*MetAP1c, inhibits bacterial growth *in vitro* [57]. *Mt*MetAP1c is inhibited at high methionine concentrations and it could not be essential. In contrast, *Mt*MetAP1a is not inhibited by methionine and it could have an essential role in methionine salvage [58]. On the other hand, in contrast to *Mt*MetAP1a, *Mt*MetAP1c retains 60% activity at pH 5.5, suggesting a major role in acidic environments, like the host macrophage phagosome [59]. Overexpressed *Mt*MetAP1a and *Mt*MetAP1c in *M. tuberculosis* confer resistance to the antibacterial effect of MetAP inhibitors [57], indicating that *Mt*MetAPs may be promising targets for the development of antituberculosis agents.

### M24 MetAP from the parasite *Leishmania donovani*

The treatment of *L. donovani* promastigotes with miltefosine (an oral drug against the parasite) induces the overexpression of the parasite M24 MetAP (*Ld*MetAP2) by 3.5 times [60]. This treatment produces an apoptotic programmed cell death with activation of caspase 3/7 protease like activity [61–63]. However, the treatment with the MetAP2 inhibitor TNP-470, or miltefosine and TNP-470, or miltefosine and the caspase-3 inhibitor N-Acetyl-Asp-Glu-Val-Asp-al, do not show activation of this activity. Moreover, MetAP2 inhibitors prevent the induction of nuclear apoptosis in *L. donovani*, as was confirmed by flow cytometry, and analysis of DNA fragmentation, translocation of phosphatidyl serine from the inner to the outer side of plasma membrane, mitochondrial membrane damage and concentration of cytosolic calcium. However, *Ld*MetAP2 inhibition does not prevent parasite cell death, since this aminopeptidase is also involved in the removal of N-terminal methionine from the nascent polypeptides [63].

The main biological functions and molecular properties of these enzymes that support them as targets are presented in **Table 1**.

## CONCLUSION

Some metalo-aminopeptidases, as *Mt*MetAP1a, PfA-M1, PfA-M17 and PfA-M18, are essential enzymes for their microorganisms and, therefore, they have been well validated as targets. Others, as *Mt*MetAP1c, *Sa*M17-LAP, PhpA, *Vc*PepA, *Tb*LAP-B, *Ac*LAP and *Tg*LAP, are not essential but are required for virulence and pathogenesis, or their activities confer some advantage for microbial growth under given conditions. For another group, formed by *Mt*LAP, *Td*M17-LAP, *Hp*M17AP, LAP-B and *Ld*MetAP2, their biological functions are predicted as crucial for microorganism survival in the human host, although they are not yet validated as targets. Some bacterial LAPs, such as *Sa*M17-LAP, *Td*M17-LAP and *Hp*M17AP, have also cysteinyl-glycinase activity. The roles of several metalo-aminopeptidases, as PhpA and *Vc*PepA, do not depend on their enzymatic activity. More work with potent and specific inhibitors or gene knockout experiments are required to elucidate the essential roles or not of these enzymes inside microbial cells. As a group, these enzymes are novel drug targets for the treatment of human infectious diseases.

## Abbreviations:

AcLAP: – A. castellanii M17 LAP;
HpM17AP: – H. pylori M17 LAP;
LAP: – leucyl-aminopeptidase;
LAP-B: – Leishmania spp. M17 LAP;
MetAP: – methionyl aminopeptidase;
MtMetAP: – M. tuberculosis MetAP;
PfA-M1: – P. falciparum M1 alanyl aminopeptidase;
Pfa-M17: – P. falciparum M17 LAP;
Pfa-M18: – P. falciparum M18 LAP;
PhpA: – P. aeruginosa M17 LAP;
SaM17-LAP: – S. aureus M17 LAP;
TbLAP-B: – T. brucei M17 LAP;
TgLAP: – T. gondii M17 LAP;
VcPepA: – V. cholerae M17 LAP.
